# Cognition from life: the two modes of cognition that underlie moral behavior

**DOI:** 10.3389/fpsyg.2015.00362

**Published:** 2015-04-21

**Authors:** Tjeerd C. Andringa, Kirsten A. Van Den Bosch, Nanda Wijermans

**Affiliations:** ^1^Artificial Intelligence and Cognitive Engineering, University of GroningenGroningen, Netherlands; ^2^Special Needs Education and Youth Care, University of GroningenGroningen, Netherlands; ^3^Stockholm Resilience Centre, Stockholm UniversityStockholm, Sweden

**Keywords:** autopoiesis, enactivism, morals, intelligence, sustainability, resilience, understanding, wisdom

## Abstract

We argue that the capacity to live life to the benefit of self and others originates in the defining properties of life. These lead to two modes of cognition; the coping mode that is preoccupied with the satisfaction of pressing needs and the co-creation mode that aims at the realization of a world where pressing needs occur less frequently. We have used the Rule of Conservative Changes – stating that new functions can only scaffold on evolutionary older, yet highly stable functions – to predict that the interplay of these two modes define a number of core functions in psychology associated with moral behavior. We explore this prediction with five examples reflecting different theoretical approaches to human cognition and action selection. We conclude the paper with the observation that science is currently dominated by the coping mode and that the benefits of the co-creation mode may be necessary to generate realistic prospects for a modern synthesis in the sciences of the mind.

## Introduction

Humans have a moral capacity to live life to the benefit of self and others. The question we address in this paper is “where does this capacity originate from and what are its defining features?” We argue that “the capacity to live life to the benefit of self and others” is a direct consequence of the defining properties of life that originated when individuals in overlapping habitats became to exist. In fact, we argue that the main constraints on behavior – and with that much of psychology – originated in the defining properties of life itself. This paper investigates the features of these essential “sub-psychological” or “pre-neural” roots and uses them to reinterpret results related to selection of behavior. As a whole the paper aims to provide a novel and productive framework to address issues related to how agents – whether human, animal, or artificial – decide on their behavior in an open world and outside the confines of controlled environments such as laboratories. In addition, we show that our framework provides, conform the call for papers, prospects for a modern synthesis to the sciences of the mind.

A main message of this meta-theoretical paper is that the definition of agentic life leads to two modes of cognition: a ‘coping mode’ and a ‘co-creation mode.’ The coping mode exists to address pressing needs and is a way to survive on the short term. The co-creation mode is prominent whenever all pressing needs are satisfied. It exists to explore the opportunities of the habitat and co-creates an environment in which the emergence of pressing needs becomes less likely. Co-creation requires agents to take both the long term as well as an extended spatial environment into account. We argue that human morality originals from the contributions of these two modes. Individuals in the coping mode are preoccupied with their very own existence and as such they may become locally oriented, short-term “egocentric” sources of potentially destructive, yet immediately self-saving, behavior. On the other hand individuals in the co-creation mode are concerned with the overall quality and dynamic stability – resilience – of the Umwelt (von Uexküll, 1992) and the possible futures it entails for them and others. As such they benefit from others in the co-creation mode and they promote the reduction of the number of connected individuals who are in or are likely to slip into the coping mode. We argue that these two modes of cognition are not only the roots of moral behavior, but also define the dynamic that stabilizes the whole biosphere.

### Paper Structure

The development of ideas in this paper is as follows: we will conclude the introduction with an important postulate – the Rule of Conservative Changes – that we use to justify the continuity between the definition of life and modern humanity.

In the Section, “Cognition from Life,” we outline a number of key concepts of the enactive approach to cognition (Thompson, 2007) that forms the theoretical underpinning for the two basic modes of cognition that we identify: the ‘coping mode’ and the ‘co-creation mode.’ Together these define ‘core cognition’ (see **Figure 2**).

In the Section “Unicellular Cooperation Virtues,” we start with connecting the roots of human morality by assuming that groups of unicellular individuals can have varying fractions of individuals in the coping and the co-creation mode. Cooperation and interaction may play out differently given the (combination of) different modes.

In the Section “Human cognition from Life” we skip a number of billion years and we scale-up the number of cells in the organism by a factor of 10^14^. At the same time we predict, on the basis of the Rule of Conservative Change, that nothing fundamental (“essential”) has changed: humans implement core cognition just as bacteria do. We explore this prediction with five examples reflecting different theoretical approaches to human cognition: (1) how the cerebral hemispheres understand the world, (2) how theories of ‘dual type processing’ of higher cognition relate to the two modes, (3) the origin of concepts such as power and wisdom, intelligence and understanding, and authority, (4) the unicellular cooperation origin of the 2+3 structure preserved in human moral values, and (5) we link the structure of positive emotions to the logic of the co-creation mode.

We conclude with the section “Prospects for a ‘Modern Synthesis’ in the Sciences of the Mind,” stating that a search for unity in science should start with unity of existence. A prominent role for the co-creation mode in science allows to progressively specify, adapt, and enrich this unity more and more by encompassing evermore theories and phenomena.

### Meta-Theoretic Departure Point

The Rule of Conservative Changes (Ghysen, 2003) states that in evolution new functions scaffold on older functions and as such preserve the essential from the very beginning. It represents the essence of our argument to connect the definition of life to moral and political behavior. Ghysen formulated the Rule of Conservative Changes as a necessary consequence of the complexity of the developmental programs, both ontogenetic and phylogenetic, of evolution ^1^. The very complexity of the developmental programs demands that the basic infrastructures on which evolutionary more recent functions rely must be “extremely stable so that they can withstand substantial variation without collapsing.” According to Ghysen:

The rule of conservative changes states that only those changes can be tolerated, that change essentially nothing. This rule applies to any set of interacting elements, where changes in any one component will alter all the interactions in which this component is involved, and adversely affect the function of the entire set. The stringency of this rule will obviously increase with the number of interactions, as it becomes more and more unlikely that a single change in one element can improve, or at least not harm, the result of the total sum of all interactions.

The rule in its most stringent form entails that whatever set of functions that initially determined what is good or bad for life must be conserved throughout evolution: it only tolerated changes that “change essentially nothing.” Yet, the same strict application of the rule will guarantee a very stable basis for innovations to rely on. Consequently, if the Rule of Conservative Changes applies, our moral values – suitably formulated – should reflect what is good or bad for life and have a stable, evolutionary old, basis. We will show that the structure of the unicellular level “morals” is still reflected in the structure of human moral virtues as formulated by Haidt (2007). Of course, a few billion years of evolution have allowed humans to come up with extremely intricate and convoluted ways to “change essentially nothing.”

Ghysen’s Rule of Conservative Changes imposes extreme stability constraints on the set of foundational older functions and the rule demands that new capacities help to improve the execution of older functions (while changing essentially nothing). However, it provides neither a starting point nor a direction. The starting point we will use here is the definition of life as formulated in the field of autopoiesis (Maturana and Varela, 1991). To impose limits on the direction of life’s development, we will use the enactive cognition approach (Di Paolo and Thompson, 2014). As such we build on the idea that the very notion of life, or more precisely living agency, already defines many of the properties of mind and our capacity to act in the world (Thompson, 2007). This paper follows up on the suggestion of Froese and Ziemke (2009) who conclude:

In order to develop a better theory of the biological roots of intentional agency we first need to gain a better understanding of bacterium-level intelligence. Only by returning to the beginnings of life itself do we stand any chance of establishing a properly grounded theory of intentional agency and cognition.

## Cognition from Life

This section builds on the Enactive approach to cognition (Varela et al., 1993; Thompson, 2007; Froese and Ziemke, 2009; Di Paolo et al., 2010). The enactive approach to cognition is based on the premise that cognition depends constitutively on the living body, understood as an autonomous system operating in a complex open environment. The enactive approach is based on concepts like autonomy, embodiment, sense-making in an environment and the activities it comprises, and the emergence of functions and behaviors originating from the interactions between the individual and its environment (Di Paolo et al., 2010).

The core of the paradigm is probably most succinctly summarized by the phrase “being by doing” (Froese and Ziemke, 2009). Consequently, for an enactivist, a system is cognitive if its behavior sustains its existence; a notion that we will take quite literally in this section. This section addresses a number of core concepts of the enactive approach (autopoiesis, viability, agency, behavior, needful freedom, adaptivity) and, if necessary, reformulates, or reinterprets them in such a way that they can be used in a wider context while still be applicable in the original context. In addition, we separate two modes of cognition: one in which behavior sustains existence in the long run and one that protects existence in times of adversity. Together these two modes address existential needs in both the long and short term.

### Autopoiesis: Needs, Identity, and Normativity

*’‘Autopoiesis’* (from Greek meaning “self creation” or “self-production”) refers to a system capable of regenerating and maintaining itself. The term was introduced in 1972 by Chilean biologists Humberto Maturana and Francisco Varela to define the self-maintaining chemistry of living cells (Maturana and Varela, 1991). Autopoiesis refers to:

A network of processes of production (synthesis and destruction) of components such that these components:

(1) Continuously regenerate and realize the network that produces them, and
(2) Constitute the system as a distinguishable unity in the domain in which they exist.

Thermodynamic constraints demand that a living self-maintaining system is far from equilibrium; consequently it requires a continual supply of energy. The moment the system looses its self-maintaining character, for example because it can no longer maintain its energy supply, it dies and eventually becomes an indistinguishable part of the environment. But as long as it is alive, autopoiesis necessarily also implies (Paolo, 2006):

(1) The establishment of a distinct “self” for which being is its own doing and with physical and organizational distinctions between inside and outside,
(2) An entity which is in constant environmental challenge, is in need of material turnover and with the freedom to achieve it, and
(3) The establishment of a normativity following the logic of metabolism according to which otherwise neutral events, both internal and external, can be good or bad for the continuation of the organism.

This implies the emergence of a “self” as a living entity that is constantly challenged by its environment, for which the events that influence it can now be evaluated in terms of facilitating or hindering its continuation. With the “self” comes a unique perspective or viewpoint, which implies for each living individual a unique history, a unique perspective, and a unique way to ensure its continuation. In short: with life comes an identity, the need for material throughput, and norms about what is good or bad in terms of consequences for the identity’s continued existence.

### Agency and Behavior

However, an autopoietic entity, although autonomously responsible for its own self-constitution, can still be limited to a fixed or externally controlled dynamic over which it has no control. As such, it may be unable to co-determine the conditions in which is exists. For co-determination, the entity needs to take control over the way it interacts with its environment: it needs ‘*agency.*’ Barandiaran et al. (2009) define an agent as:

An autonomous organization that adaptively regulates its coupling with its environment and contributes to sustaining itself as a consequence.

Being agentic, or not, corresponds to being a passive recipient of environmental challenges or to (pro-)actively controlling and selecting these environmental challenges (Paolo, 2006). Only the second mode of interaction fully deserves the name ‘*behavior*’ (Paolo, 2006), because it is the agent that regulates its relation to the environment. This agent-controlled regulation of the coupling with the environment gives the organism whole new levels of freedom to continue its existence. We will refer to his strategy as ‘living agency’ (or ‘agency’ for short).

### Needful Freedom

The relation between a living organism, as a dynamically maintained material structure, and the matter on which it depends, leads to a form of existence that has been called ‘*needful freedom*’ (Froese and Ziemke, 2009):

This relation is best expressed through the fact that, while the existential form of an organism is independent of any particular configuration of matter through which it passes in virtue of its metabolism, it is nevertheless dependent on the continual perpetuation of this ongoing flow of material configurations. If this flow ceases and the organic form fully coincides with its current material configuration then it is no longer living.

Formulated like this, *life is about need satisfaction*: as long as an entity exists that has the need for an ongoing material throughput and sustains this throughput itself, it is alive and viable. The moment the ongoing flow cannot be sustained, the entity becomes, again, part of its environment and looses its identity.

Needful freedom allows living agents the liberty to engage with its environment in any of a multitude of ways to satisfy its metabolic needs. This is the basis of the individual’s autonomy and freedom that Di Paolo (2009) describes as follows:

The fact that metabolism sustains a dynamic form of identity (not coinciding with its material constitution at any given time except at the time of death) allows an organism to become free. This freedom is expressed in the capability of the organism to engage with its medium in terms of the significance of a situation, thus contributing to its continuing dynamical autonomy and even opening up the possibility of novel value-making. However, this freedom is allowed by very strict and specific material needs. It is a needful freedom.

Needful freedom severely constrains behavior, because viability may never become zero. The organism should in fact always aim to remain as viable as possible, because close to the viability boundary (e.g., at birth or death) it has more pressing and more specific material needs and is even more dependent on the particulars of its immediate environment to satisfy its immediate needs.

This suggests the need for two complementary sets of need satisfaction strategies. One set to effectively satisfy *particular* material needs and another set to create the conditions in which *all* needs can be satisfied as well as possible. Both strategies give existential significance – meaning – to the particulars of the (immediate) environment. This process of meaning-giving or sense-making forms the basis of the individual’s uniqueness, because each individual exists in a (slightly) different environment and must create a unique history of strategies to engage it. This history of activities also provides the individual a unique learning history to benefit from.

### Two Modes of Cognition

Within the enactive approach the terms ‘cognition’ and sense-making are equated with autopoietic performance (Maturana and Varela, 1991). For example Di Paolo and Thompson (2014) conclude:

Cognition, in its most general form, is sense-making—the adaptive regulation of states and interactions by an agent with respect to the consequences for the agent’s own viability.

Within enactivism, cognition is not so much a function but an ongoing process of sense-making: valuing the opportunities of the environment in terms of contributions to an organism’s continued existence. If at all possible, this cognitive process must lead to the creation of conditions of sustained high viability due to successful long-term need satisfaction.

This leads to a definition of cognitivity (Di Paolo and Thompson, 2014):

A system is cognitive when its behavior is governed by the norm of the system’s own continued existence and flourishing.

This definition of cognitivity suggests two modes of cognition. The first mode is governed by “the norms of the agent’s continued existence” and corresponds to what we will refer to as the ‘coping mode of cognition’ because it is aimed at the *satisfaction* of—pressing—‘deficiency needs.’ The second mode of cognition is aimed at *preventing* pressing needs, while being “governed by the norms of the agent’s flourishing,” and will be referred to as the ‘co-creation mode of cognition.’

Cognitivity is defined for the domain of complex systems (Kauffman, 1995): systems characterized by many interacting entities (Strevens, 2006), the absence of central control, and long-term system inpredictability. The co-creation and the coping mode have different scopes and objectives that correspond to the difference between ‘resilience’ and ‘stability.’

Resilience determines the persistence of relationships within a system and is a measure of the ability of these systems to absorb changes of state variables, driving variables, and parameters, and still persist. Stability, on the other hand, is the ability of a system to return to an equilibrium state after a temporary disturbance (Holling, 1973).

The resilience of a system leads to a persistence of relationships that allow cognitive agents to rely on the overall dynamics of that system. By enhancing beneficial over detrimental relationships, the co-creation mode can set-up the conditions for its continued existence and flourishing. The scope of co-creaction is therefore holistic, because it involves all aspects and all timescales of agent and environment. The coping mode in contrast aims for the return of *particular* equilibrium states in the agent (basic need fulfilled) and its scope is limited to what is necessary for the realization or maintainance of these stable states when required. While the coping mode is ‘reactive,’ the co-creation mode of cognition is ‘proactive.’

Resilience can be defined at many levels of description and the concept has many different domain specific definitions (Brand and Jax, 2007). In our case (a) the level of the individual – for humans ego-resilience (Cohn et al., 2009) – and (b) the social ecological system (Walker et al., 2004; Folke et al., 2010) it is part of, are the most relevant. In particular, resilience as defined as *“the capacity of a system to absorb disturbance and reorganize while undergoing change so as to still retain essentially the same function, structure, identity, and feedbacks”* (Walker et al., 2004) is appropriate. Note that we could also refer to the co-creation mode of cognition as “resilience build-up” or as ‘being cognition’ (Maslow, 1962, 1963), because it sets up the conditions for successful *‘being.’* Equally the coping mode could have been referred to as ‘deficiency cognition’ (Maslow, 1962, 1963).

### Long Term Viability

**FIGURE 1:**
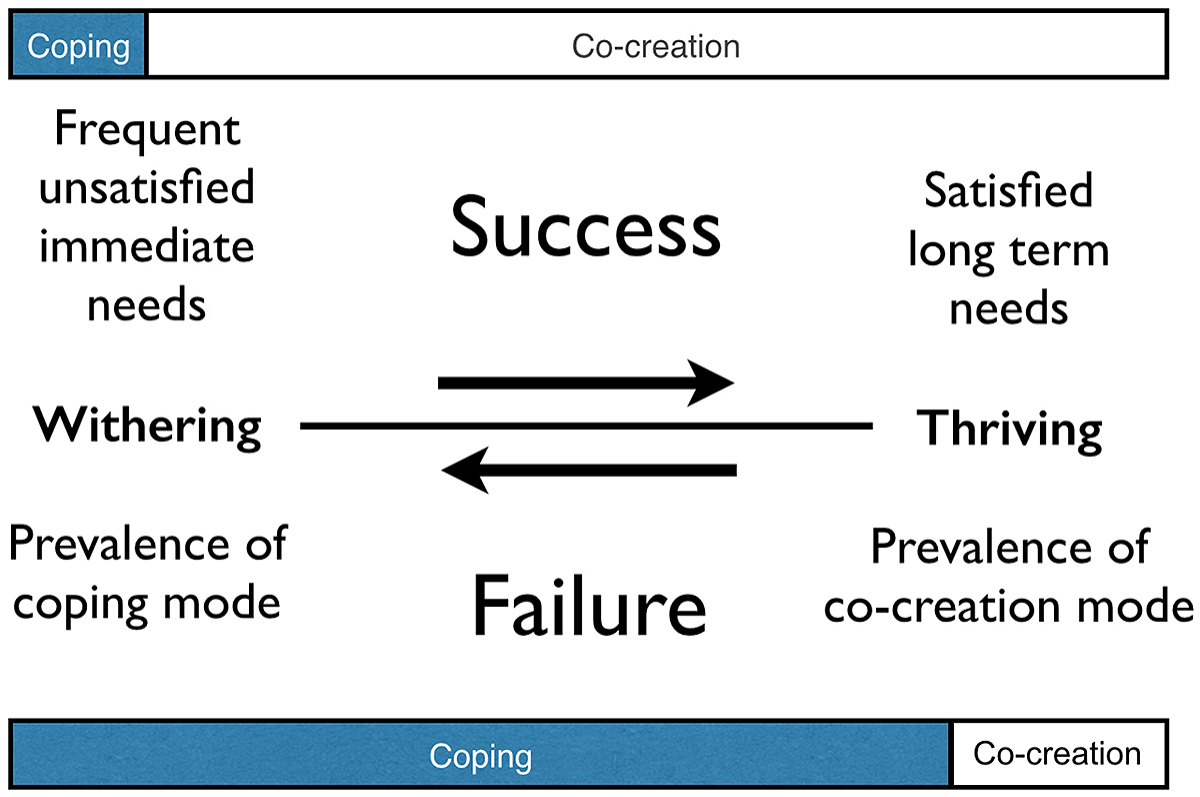
**Long-term viability measure. The more prevalent the co-creation mode, the higher autopoietic quality**. The states on the right correspond to thriving and the states on the left to withering.

The definition of cognitivity leads to a long-term viability measure: high quality autopoietic performance entails satisfied long-term needs, while low quality autopoietic performance is apparent as frequent or continually pressing immediate needs. The more a system is in the co-creation mode, the higher its viability and vice versa. This quality measure is depicted in **Figure 1**.

Note that this is a quality measure related to well-being (in terms of satisfied needs) and not directly to fitness or evolution (the theory of autopoiesis defines life, not the strategies life has found to remain alive). However, it is safe to assume that high well-being is conducive for the generation of healthy and procreating offspring. Also, the average lifespan of individuals who regularly approach the boundary of their viability, i.e., are in mortal danger, will be lower, just as their window to procreate.

### Adaptivity and Sustainability

Both modes of cognition, coping, and co-creation, are concerned with the prevention of an adverse future and are, as such, reflections of the essentially anticipatory nature of life (Vernon, 2010). The main proponent of the central role of anticipation in biology was Robert Rosen who defined anticipatory systems as follows (Louie, 2010):

An anticipatory system is a natural system that contains an internal predictive model of itself and of its environment, which allows it to change state at an instant in accord with the model’s predictions pertaining to a later instant.

Cognition then relies on an internal predictive model (also Vernon, 2010), involving the relations between self and environment, for the identification of viability impacting likely future states and the development of a decision strategy to select a beneficial future state while avoiding detrimental future ones.

This demand is covered by the term ‘*adaptivity*’ (Di Paolo, 2009) for, in particular, the coping mode of cognition of autopoietic systems. Di Paolo defines adaptivity as follows:

Adaptivity: A system’s capacity, in some circumstances, to regulate its states and its relation to the environment with the result that, if the states are sufficiently close to the boundary of viability,

(1) Tendencies are distinguished and acted upon depending on whether the states will approach or recede from the boundary and, as a consequence,
(2) Tendencies of the first kind are moved closer to or transformed into tendencies of the second and so future states are prevented from reaching the boundary with an outward velocity.

In this definition tendencies refer to likely futures. Interestingly, this definition only distinguishes tendencies if they are sufficiently close to the boundary of viability, which suggests that adaptivity is only needed in situations of immediate danger. However this does not cover the conditions in which the system flourishes.

By adding a third component to Di Paolo’s definition of adaptivity, the co-creation mode of cognition is also covered. This leads, after a slight reformulation in italic, to a new concept that we will call ‘*sustainability*’ (of self and the environment).

Sustainability: A system’s capacity, in some circumstances, to regulate its states and its relation to the environment with the result that

(1) *Anticipation:* Tendencies are distinguished and acted upon depending on whether the states will approach or recede from the boundary of viability.
(2) *Coping: If the states are sufficiently close to the boundary of viability,* tendencies of the first kind are moved closer to or transformed into tendencies of the second and so future states are prevented from reaching the boundary with an outward velocity.
(3) *Co-creation: If the states are sufficiently far from the boundary of viability, tendencies of the second kind are used to create an ever more spatially and temporally extended environment for proactive need satisfaction.*

Sustainability, defined as such, complies with and even extends the usual use of the term because it is a recipe not just to *conserve*, but also to actually *create*, a stable and reliable ecological dynamic. Applied to a global scale with overlapping and structurally interwoven habitats, it implies the Gaia hypothesis (Lovelock and Margulis, 1974), which proposes that all organisms and their inorganic surroundings on Earth are closely integrated to form a single self-regulating complex system that maintains the conditions for life on the planet (i.e., life itself sets up the conditions for its proactive need satisfaction). This dovetails with Margulis and Sagan (1995) who wrote:

“Darwin’s grand vision was not wrong, only incomplete. In accentuating the direct competition between individuals for resources as the primary selection mechanism, Darwin (and especially his followers) created the impression that the environment was simply a static arena.”

Indeed, the competition (coping mode) takes place in a complex environment continually maintained and co-created by life for its own benefit (co-creation mode).

The three aspects of the definition of sustainability – anticipation, coping, and co-creation – deserve some more attention.

### Anticipation: Original Perspective

Any agent develops a history of activities by which it is at any instant constrained, so it always builds on its earlier activities. Or put differently: it is for better or for worse, always confronted with the consequences of its own actions. By selecting its activities well, i.e., timing its behaviors well, the agent can, at least for some time, avoid states it cannot handle and select or co-create states that allow it to thrive. Consequently, the anticipatory nature of successful autopoiesis requires the prediction of possible viability developments through some model of itself in relation to the environment.

Since the simplest predictive model is only based on the aggregate of internal and external states, the earliest perception-action models were based on the aggregate of internal and external influences and were therefore unable to separate these. On top of this “holistic” evaluation more advanced perceptual mechanisms have evolved that, eventually, could separate internal from external influences (including influences from other agents). One essential property of this ‘original perspective’ is that it is holistic and context sensitive. This theoretical consideration will be applied a number of times in the rest of this paper.

The original perspective was not only holistic and context sensitive, but also essentially subjective: it was both individual and deeply value-laden in terms of whether it reflected tendencies that approach or recede from the *individual* boundary of viability. This can be interpreted as a perspective on the safety of the individual that it in part should learn through exploration (a form of participation in the environment). The development and initiation of appropriate (tendency transforming) activities depend therefore on the individual’s history and are unique for the individual.

This (again) entails that each individual is its own sense-maker in terms of how it interprets tendencies as beneficial (good), detrimental (bad), or irrelevant, depending on whether they recede or approach the boundaries of viability. Yet, although each individual is its own sense-maker, it is also a member of a species and it shares many essential aspects with other life forms, entailing the existence of general sense-making strategies. These commonalities form the basis for morality defined as “the extent to which an action is right or wrong” (New Oxford Dictionary) on which we will build.

### Coping

In situations that are experienced as indicative of immanent danger of viability loss, the agent is confronted with one or more unsatisfied needs as pressing problems to address; even if this goes at the cost of other aspects that are currently not critical. The coping mode prioritizes and as a result is focused and sequential. Coping favors the certainty of control over improvisation and as such the autopoietic system will tend to keep or bring all essential parameters within the bounds of normal functioning, using whatever reliable utility (in- or external) it has access to. This entails that the coping mode of cognition is essentially conservative: to protect the essential, it will sacrifice the unessential and/or currently worthless as an inevitable side effect. Concepts that aptly describe the functioning of the coping mode are ‘trying to control the situation,’ ‘reactive problem solving,’ ‘prioritizing,’ ‘conservation of the essential,’ ‘short-term utility for self-preservation,’ and ‘acceptance of adverse side effects.’

Following Di Paolo’s (2009) ‘adaptivity,’ life on earth started as a perpetual “reactive struggle” that could only exist in the most favorable conditions. Life could become only stable and comfortably established when the aggregate of living individuals succeeded in *proactively* co-creating and maintaining—eventually earth-wide—the conditions for their own existence: life extended ‘adaptivity’ to ‘sustainability.’ This line of reasoning suggests that the coping mode has older evolutionary origins and that the co-creation mode evolved as a safer strategy by ever-expanding the “scope of normality.”

### Co-creation

The need to activate the coping mode is indicative of a failing or inadequacy of the co-creation mode. Apparently the agent failed, through its own fault or not, in proactively maintaining a situation without pressing needs, which forced the coping mode to reactively solve the problem. Since the coping mode of cognition is essentially a fallback in case of a failing co-creation mode—with the ultimate objective to preserve agency or life by conserving the *essential* at the cost of the currently not essential and bringing it back within the scope of normality—the core task of the co-creation mode is to restore the *overall* functioning of the system, and to consolidate the whole system after insult.

After the autopoietic system has consolidated itself and is fully viable again, the priority shifts back to co-creation to prevent new insults or to come up with ways to mitigate their effects proactively to optimize the long-term viability of the autopoietic system in its environment. Thus the co-creation mode builds on the holistic and context sensitivity of the original perspective. Concepts that describe the co-creation mode are ‘prevention of problems,’ ‘holistic optimization,’ ‘context sensitivity,’ ‘consolidation after repletion,’ and – as much as possible – the ‘creating and maintaining of a safe and sustaining environment with long-term benefits.’

This suggests a way to introduce concepts like ‘good’ and ‘bad’ in terms of resilience. A ‘good’ influence increases *“the capacity of a system to absorb disturbance and reorganize while undergoing change so as to still retain essentially the same function, structure, identity, and feedback”* (Walker et al., 2004) while a ‘bad’ influence erodes, or in extreme cases destroys, this capacity. In fact we can call tendencies that move agents to the left in **Figure 1** ‘bad’ and tendencies to the right ‘good.’ Note that what is good for a system defined on one aggregation level can be bad on another (and the same with short and long term).

### Summary of Cognition from Life

The subsection above discussed that a living agent decides, in part, on its own future via behavior that selects advantageous reachable future states according to its own norms and on the basis of some sort of predictive model that optimizes future viability. Cognition boils down to the selection and execution of activities promoting the individual’s continued existence and flourishing, which encapsulates the enactive approach as “being by doing” (Froese and Ziemke, 2009). We propose two modes of cognition: a coping mode of cognition focused on keeping a living agent in mere existence, and a co-creation mode of cognition focused on the flourishing of a living agent. Together, we will refer to these two modes and the concept that emerge from them as ‘core cognition.’

**FIGURE 2:**
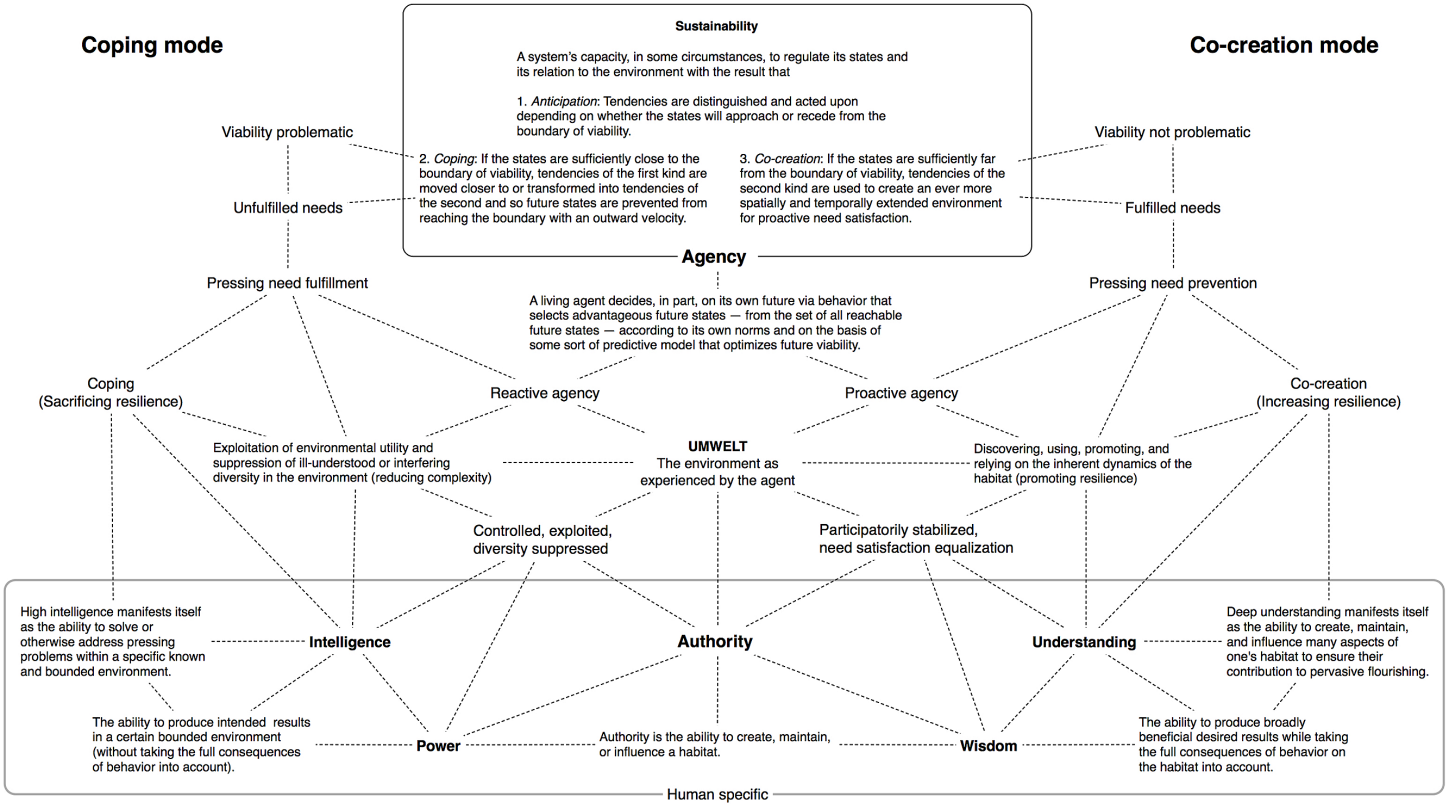
**Concept map of core cognition**. All concepts emerge from ‘sustainability’ and related concepts branch out left (coping mode) and right (co-creation mode). The concepts in the middle are independent of and interpreted differently in the coping and the co-creation modes. The concepts in the block at the bottom are often seen as uniquely human and will be addressed in Section “Human Cognition from Life.”

**Figure 2** represents a visual summary (concept map) of ‘core cognition.’ The definition of sustainability is the “starting point” (top-middle). From there contrasting, but not necessarily similar or complementary, consequences of coping (left) and co-creation (right) branch out to the side and below. Both the concepts ‘agency’ and ‘Umwelt’ are common for the two modes. The concepts in the lower square (‘authority,’ ‘intelligence,’ ‘understanding,’ ‘power,’ and ‘wisdom’) are typically attributed to human cognition and emerge quite naturally and without the need for intermediate steps or levels, which will be discussed in Section “Human Cognition from Life.”

## Unicellular Cooperation Virtues

In this section, we remain at the unicellular level, but unlike Section “Cognition from Life” we focus on relations between individual living agents. In particular we address cooperation from the viewpoint of the coping and the co-creation mode. We first discuss the need for cooperation, then the resulting group perspective, and finally a number of unicellular cooperation virtues. We will compare these virtues with human moral values in Section “Haidt’s Moral Virtues.”

### Unicellular Cooperation

One essential activity of living agents with important consequences is procreation. Unicellular organisms procreate by dividing and thus end up as neighboring individuals. After a number of generations in favorable situations – conditions in which all needs are satisfied and therefore indicative of the co-creation mode – this results in many individuals in overlapping habitats. This success leads, inevitably, to problems associated with the autopoietic demands for material throughput: a growing demand (per volume) for nutrients and energy and more waste products to dispose of. Yet, it makes cooperation possible. Sociality is therefore both a challenge and an opportunity for life and as such it offers the possibility for a self-stabilizing dynamic.

As stated before, the predictive models of the most primitive unicellular life forms are holistic and unable to separate in- and external states. Individuals of early life forms based activities on holistic predictive models that account for the state of the individual in its environment (whether social or not). As a consequence, sociality does not require qualitative different decision processes compared to “individual-level decisions.” As long as the predictive model can learn to select advantageous strategies, given the environment, it will serve the individuals (and the species alike).

However, what is advantageous differs between the co-creation and the coping mode. The co-creation mode favors *prevention* of problems, consolidation after repletion, and the creation and maintenance of a safe and sustaining environment with long-term benefits. In contrast, the coping mode involves increasing *control* over the situation, to prevent one from becoming an inadequate or even dead agent. The coping agent does this through conservation of the essential, exploiting short-term utility for self-preservation, and ignoring or accepting adverse side effects in the course of pressing need satisfaction.

Based on the composition one can imagine three types of groups: all resilient, all coping, or a mixture of individuals in the co-creation and the coping mode. When *all* individuals are in the co-creation mode (which might not often be realistic) this can result in a combination of cooperation and individual or group-wise exploration with the creation and maintenance of an environment that is as safe and sustaining as possible. If, however, *all* individuals are in the coping mode, this may lead to a relentless competition between opportunistic individuals with as consequence the survival of the “fittest” (actually the survival of those that cope/compete best given the environment). Or alternatively, they can cooperate conform the strengths of the coping mode and engage in highly regimented behaviors that may be very effective in addressing the (now) shared needs. However, unlike the co-creation mode this behavior is not necessarily without adverse side effects.

In situations with individuals in both modes, the balance between cooperation and self-enhancing benefits determines the outcome. For example, Cremer et al. (2012) observes a typical three phase repetitive cycle of bacterial population dynamics. The cycle starts with relatively few and independent bacterial cells that do not procreate. In procreation promoting conditions, individuals form groups according to chance. These groups develop differently according to the fraction of individuals more inclined to the coping mode – who focus on pressing need satisfaction and who Cremer refers to as cheaters or defectors – versus those in the co-creation mode – who optimize the aggregate of self and the environment (cooperators). Cremer et al. (2012) observes two characteristic features of the groups’ internal dynamics:

“First because of the costs for providing the benefit, cooperators have a selection disadvantage, compared to cheaters in the same group. In particular, cooperators reproduce slower than cheaters and hence the fraction of cooperators decreases within each group (intra-group evolution). Second, considering the benefit of cooperation, groups with more cooperators grow faster and can reach a higher maximum size (carrying capacity) than groups of mainly cheaters (inter-group evolution).”

The moment the conditions for growth are no longer maintained, the groups dissolve and the individuals become again more independent, but now in new numbers of cheaters and cooperators.

We can conclude that for individuals in the co-creation mode it is highly beneficial to have as many others as possible in the co-creation mode as well, which implies that if coping is not dominant, a community of individuals can thrive. This entails that ‘caring’ behavior, which helps individuals shift from the coping mode into the co-creation mode, is a viable tactic in the co-creation mode to effectively promote overall thriving. Conversely when coping becomes dominant, thriving becomes increasingly rare. This is a rephrasing of the long-term viability measure (**Figure 1**), but now on a group level.

This example illustrates the key characteristics of the coping and the co-creation mode. The coping mode might favor unsustainable forms of cooperation, such as competition at the cost of, for example, fellow individuals who compete less well. The co-creation mode on the other hand realizes global benefits in the form of a higher carrying capacity. Both strategies have evolutionary advantages: the coping mode in times of adversity, in which a reduction of the number of individuals is actually beneficial given limited resources, and resilience enhancement (co-creation) in times of plenty. However, not all individuals may make the switch from one mode to the other at the same time and some may be more inclined to one particular strategy. Nonetheless individuals in different modes exhibiting quite different strategies can coexist.

### The Emergent Group Perspective

Cooperation leads to the emergence of group-level agency and with that to the emergence of group-level meaning giving and sense-making. It also leads to a group-perspective in addition to the perspective of each individual. Without cooperation, the associated perspective is that of the unicellular individuals and behavior is selected from the set of all reachable future states per individual. A cooperating group creates a new perspective in which behavior is selected from all reachable future states of that group, which might be quite different and, at times, even conflicting with the demands at the unicellular level.

In particular the cooperative perspective creates an aggregate or group level to which one can ascribe the coping and co-creation mode. For example

“myxobacteria are Gram-negative organisms that are capable of multicellular, social behavior. In the presence of nutrients, swarms of myxobacteria feed cooperatively by sharing extracellular digestive enzymes, and can prey on other bacteria. When the food supply runs low, they initiate a complex developmental program that culminates in the production of a fruiting body”

(Kaiser, 2003).

In this case the bacteria start with a kind of loose cooperation (allowing for a diversity of individual activities) of independent agents in times of plenty – a group level co-creation mode – that develops into a highly regimented (uniform, predictable, and coordinated) and eventually even sacrificial collaboration to produce the spores that continue the species elsewhere at a later stage – a group level coping mode.

This suggests that in the co-creation mode individuals have maximal agency and freedom to pursue individual or collective futures, which may lead to the discovery of ever more versatile cooperative or individual strategies that progressively bring more and more situations within the scope of the co-creation mode. The group level coping mode results into individuals trading “freedom for security” and engaging in highly regimented behaviors with particular need satisfaction purposes such as *“accessing resources that cannot effectively be utilized by single cells, collectively defending against antagonists, and optimizing population survival”* (Shapiro, 1998).

### Unicellular Cooperation Virtues

The factors that define the actual form of cooperation depend essentially on the scope of the cooperation benefits (for some ingroup or for all) and the degree to which the needs are pressing. A cooperating agent in the co-creation mode should not only maintain its internal network, but it must take the needs of its collaborators, as well as the overall state of the environment, into account. This quite naturally leads to a basic concern – a care – for a general well-being, including the capacity to prevent harming others, assist the suffering, and a concern for the shared environment (which it can because it is based on a holistic evaluation).

The “caring agent” will be sensitive to the needs of others and in particular be concerned about sufficient ‘need satisfaction equality’ among individuals in its Umwelt since ‘unfulfilled need inequality’ leads to diversity in behavioral strategies (co-creation versus coping) and as such to the undermining of (long-term) collaborative efforts and overall (group-level) stability. Strategies involving a shared *care* for a *fair* distribution of fulfilled (and unfulfilled) needs are highly self-stabilizing and resilience enhancing, while at the same time allowing for the discovery of new dynamically stable states.

Note that the scope of need satisfaction may not only involve ones own species, but in principle all living agents who contribute to the holistic situational awareness – Umwelt – on which action selection is based. These strategies are examples of the co-creation mode and may even underlie the emergence of Gaia as the global self-sustaining network of living entities that created and maintained the atmosphere and biosphere it relies on for its continued existence (Lovelock and Margulis, 1974).

The aggregate situational awareness characteristic of the first life forms are likely to be conserved according to the constraints imposed by the Rule of Conservative Changes. If so, this could be a basis for empathy (according to the New Oxford Dictionary “the ability to understand and share the feelings of another”): however, not limited to conspecifics, but conforming with the breadth of the holistic situational awareness, toward to the whole Umwelt. Empathy might therefore be understood in its original form as “the ability to understand and be influenced by the state of the whole environment.” We will return to the concept of ‘understanding’ in a later section, where we define it.

Yet not all collaborative strategies may, in principle, be broadly beneficial. Some may explicitly promote the satisfaction of particular ingroup needs to the detriment of other groups or individuals and the strategies are examples of the coping mode. Coping strategies need a clear ingroup/outgroup distinction so that ingroup members are recognizable, loyal, and willing to disregard or exploit outgroups. In addition all group members are expected to identify and execute group roles properly (and without error). Finally, ingroup cooperation requires the behavior of all group members to be constrained by ingroup level rationality. In particular behaviors that stem from individual-level needs (i.e., make sense from rationality constraints at the level of the individual) should be resisted if they exceed group-level norms or lead to group-level costs.

These behaviors give rise to two qualitatively different sets of standards of conduct for ‘cooperation virtues.’ The first set allows for broadly beneficial, i.e., global, cooperation through a care for all agents and their needs in the environment in combination with efforts to realize a by and large equal, or fair, distribution of satisfied needs. The second set of standards allows for effective within-group dynamics and even competition between in- and outgroups. In this mode, ingroup loyalty, ingroup role adherence, and ingroup-level rationality constraints on individual behavior are central. **Table 1** summarizes these group-level cooperation values.

**Table 1:** Cooperation virtues formulated from unicellular level cognition.

Scope of optimization Cognitive mode	Cooperation virtue	Description
Global – long term Co-creating and maintaining conditions for pervasive need satisfaction	1 – Care	Concern and shared responsibility for the need satisfaction in others in particular through preventing harm in others, assisting those in need, and care for the environment in general (promoting the co-creation mode).

Co-creation mode	2 – Fairness	Promotion of equality in terms of the level of satisfied needs to prevent a diversity of unsatisfied needs (preventing the coping mode).

Local – short term Creating and maintaining conditions suitable for effective ingroup coping	3 – Ingroup loyalty	Showing/proving you are a member of the ingroup through signification, self-sacrifice, ingroup loyalty, and disregard or exploitation of outgroups.

	4 – Ingroup role adherence	Proper identification and execution of ingroup roles and norms (prevention of mistakes), submission to ingroup consensus, or a central coordinating center.

Coping mode	5 – Ingroup rationality constraints	Self imposed limits on behavior according to ingroup-level rationality. For example resistance to pursue individual-level selfish needs that exceed ingroup norms or tempt others to exceed ingroup constraints as well.

*This table can be compared to the **Table 2** (Haidt’s moral values)*.

### Cooperation and Agency

In the co-creation mode, social individuals have maximal agency and freedom to pursue individual or collective future as long as they are, at the same time, sensitive and responsive to the needs of other agents in their Umwelt and promote more or less equal levels of need satisfaction. Within these bounds this mode may lead to the discovery of ever more versatile individual and cooperative strategies that progressively bring more and more situations within the scope of the co-creation mode and in doing so increases the carrying capacity of the environment. This results in widely shared benefits and the generation of evermore stable, reliable, and beneficial nested relationships between individuals, species, habitats, and even the global eco-system. In short: broad resilience built-up. A general state of thriving is the hallmark of the success of the co-creation mode. This form or cooperation relies essentially on individual-level agency as a resource.

In the coping mode (e.g., when food supply runs low) social individuals may choose to trade “freedom for security” in which they engage in highly regimented collaborative behavior with a particular need satisfaction purpose and particular stable states. This form of cooperation treats individual level agency as a stability threat that should be curtailed instead of stimulated. In this mode individuals treat the environment as a resource and a buffer of utilities to be exploited. More constructively, it can also lead to a particular form of constructive cooperation intended to benefit the ingroup (possibly at the costs of outgroups). This form of cooperation relies essentially on ingroup-level agency as a resource.

For later reference, the previous can be summarized as follows:

*Global versus Local Optimization* Caring for all (global) versus caring for oneself or for a particular ingroup (local) relates directly to cognitive modes that drive behavior and thus determine the type of cooperation. Agents in the co-creation mode engage in long-term optimization of the opportunities and dynamic stability – resilience – of the combination between self and the environment. Their cooperation, involves all agents in the context of their Umwelt. To do so cooperating individuals should be generally caring and promoting equality of need satisfaction levels. On the other hand, unsatisfied needs and inequality in need satisfaction levels promote a prevalence and diversity of individuals in the coping mode. These are motivated by short-term, small-scope, ingroup, and situation specific goals.

We will return to this summary in the next section when we address moral virtues.

## Human Cognition from Life

As we suggested in the Introduction, we will now skip a few billion years and scale-up the number of cells of the organism by 14 orders of magnitude. We assume, on the basis of the Rule of Conservative Changes, that *nothing fundamental (“essential”) has changed*, entailing that both unicellular and human behavior can be described by the two cognitive modes, i.e., the coping and co-creation mode that define core cognition. In this section we substantiate this, at first glance quite extraordinary prediction, with five examples of well-known phenomena and theories reported in modern psychology that all pertain in some way to “the capacity to live life to the benefits of self and others.”

We consider these clear examples of modern day manifestations of core cognition (the upper part of **Figure 2**) and in particular of the long-term viability measure (**Figure 1**), the co-creation and coping mode, and unicellular level cooperation virtues (**Table 1**). The given examples build on each other and are intimately related because they are manifestations of core cognition. We will address:

(1) The bihemispheric structure of the brain (McGilchrist, 2010) implementing two attitudes toward a complex world.
(2) Dual type processing in relation to the coping and the co-creation mode.
(3) The derivation of the concepts of ‘intelligence’ and ‘power’ from the properties of the coping mode and the concepts of ‘understanding’ and ‘wisdom’ from the co-creation mode.
(4) The interpretation of the structure of human (strictly speaking American) moral values as straightforward extension of unicellular cooperation values (Haidt and Graham, 2007).
(5) The broaden and build theory of positive emotions (Fredrickson and Branigan, 2005; Cohn et al., 2009) reflecting key properties of the co-creation mode.

In a recent paper called “Learning autonomy in two or three steps: linking open-ended development, authority, and agency to motivation” (Andringa et al., 2013), we already combined many of the key concepts in these five examples. The Learning Autonomy paper focused on the development of human cognition and autonomy during a life span (ontogenesis). The present paper addresses the evolution of cooperative behaviors of individuals in groups, and groups in an environment (the phylogenesis of behavior). The present paper thus allows us to understand *why* the concepts emerged in Learning Autonomy the way they did. We will refer a number of times to that paper. Together – combining ontogenesis and phylogenesis – these two papers bolster our claims even further.

Note that we cannot really proof our prediction. What we aim for is to show the existence of a high degree of consistency between unicellular level cognition – core cognition – and results from modern Psychology. Consistency and similarity, even to an uncanny level, are indicative but not conclusive proof of the prediction that since the stable emergence of life nothing essential has changed and thus that the definition of life already contained the determinants of cognition. So, for the moment, it is not proof but plausibility we aim for.

### Example 1: Two Attitudes Toward the World and Two Brain Hemispheres

In Learning Autonomy (Andringa et al., 2013) we observed that *successful* life span development is characterized by an ever-improving understanding of reality in combination with an urge (and proven ability) to improve and shape the Umwelt. This fits the description of the co-creation mode that we coupled to the “prevention of problems, consolidation after repletion, and – as much as possible – the creation and maintenance of a safe and sustaining environment with long-term need satisfaction potential.” In Learning Autonomy we interpreted cognitive development (in humans and human-like artificial agents) as learning to master the complexity of the world.

Life is always near the ‘edge of chaos’ (Mora and Bialek, 2011) and if the complexity of the current situation is judged too high we benefit from coping strategies that reduce its complexity and make the situation more tractable and predictable. In Learning Autonomy we referred to the form of cognition that allows us to curtail a complex world as “cognition for order,” “cognition for certainty,” or “control cognition.” We associated this form of cognition with fear and anxiety, detachment, abstract manipulation, and the personality trait ‘closed to experience.’ This description matches with the concepts that we used to describe the coping mode: ‘trying to control the situation,’ ‘reactive problem solving,’ ‘conservation of the essential,’ ‘short-term utility for self-preservation,’ and ‘acceptance of adverse side effects.’

Yet at other moments we can deal with some additional complexity and allow ourselves to explore the possibilities of the world. Successful, typically playful and purposeless, exploration leads to the discovery of new, generic or invariant structures that make the world a bit more tractable and accessible to agentic influences. This expansion of the understanding of the world fits with the holistic nature of the co-creation mode.

In Learning Autonomy we observed that the two modes we identified matched the description of differences in the way the left and right cerebral hemispheres understand the world and contribute to our existence according to the seminal work “The Master and His Emissary” by McGilchrist (2010). Table 1 of Learning Autonomy provides an comprehensive summary of the reported differences between (and complementarity of) the attitudes toward the world associated with the left and the right hemispheres that exemplifies how the coping and the co-creation modes are implemented in modern humanity (and in particular the brains of human individuals).

McGilchrist (2010) argues that our Western societies have become characterized by an ever growing dominance of the left-hemispheric – coping – world-view that favors a narrow focus over the broader picture, specialists over generalists, fragmentation over unification, knowledge and intelligence over experience and wisdom, technical objects over living entities, control over growth and flourishing, and dependence over autonomy. Apparently, despite the huge cultural progress that has been made in the last millennia, humanity shifted more and more toward the coping mode. According to the summary in **Figure 1** this is a neither a sign of autopoietic success, nor of viability: on the contrary. Apparently, our understanding of society has not matched society’s complexity growth.

This erosion of the co-creation mode of cognition, and, directly coupled, the resilience reduction of our natural environment, may in fact explain why humanity faces a number of existential problems and in particular has difficulties in realizing a sustainable long-term future: the coping mode, with a focus on pressing problems, intolerance to diversity, and its insensitivity to adverse side-effects as key characteristics, is simply unsuitable to setup the conditions for easy and reliable future need satisfaction.

### Example 2: Dual Type Processing

The previous section may have suggested that the coping mode is an inferior mode of cognition that is mainly useful in situations where the co-creation mode is inadequate and long-term adverse side effects are the least of one’s worries. On a long-term strategic level this may be true, but in the short-term of daily mental processes we propose that the interplay between both modes allows ever-improving action selection. We do this by connecting to dual-process theories of higher cognition.

Dual-process or dual system theories of higher cognition (Evans, 2003; Evans and Stanovich, 2013) rely on the existence of two qualitatively different systems that, together, span the full scope of mental processes. These theories are still under development and not without criticism (Keren and Schul, 2009), yet they easily fit in our discourse. In a recent paper addressing this criticism Evans and Stanovich (2013) separate defining and correlative features of two types of mental processes (that each may have hemispheric biases, but that are definitely *not* exclusively associated with a single hemisphere).

According to Evans and Stanovich (2013) the defining properties of type 1 – intuitive – processes are that they are autonomous and do not require working memory, while type 2 – reflective – processes do require working memory and allow for cognitive decoupling from the here and now to allow “hypothetical reasoning and cognitive simulation” (Stanovich et al., 2011).

We summarized the Section “Cognition from Life” with the following conclusion about living agency:

A living agent decides, in part, on its own future via behavior that selects advantageous future states (of the aggregate of self and environment)—from the set of all reachable future states the agent has access to—according to its own norms and on the basis of some sort of predictive model that optimizes its future viability.

We propose that an intricate interplay between type 1 and 2 processes, a few billion years later, implements this. Type 1 processes bring and keep the agent autonomously—without central control – from the set of all possible states of reality into a mindset appropriate for the here-and-now. This mindset *presents* reality (McGilchrist, 2010; Andringa et al., 2013, Table 1) and especially its most salient and potentially meaningful or otherwise pressing aspects as Umwelt. Type 1 processes set up the stage for all action selection and are a manifestation of the original (holistic) perspective. Automatic behaviors like walking or habits like brushing your teeth rely on the autonomy and situational awareness of type 1 processing. We have partial conscious access to the outcomes of type 1 processes as a holistic experience (Kaplan, 1995), direct perception (Gibson, 1986), or as gist phenomena (Oliva, 2005).

Type 2 processes take the generated Umwelt as basis for non-automatic and non-habitual action selection to propose an even more beneficial future than automated or habitual, type 1, responses can realize. This more complex action selection process involves the comparison of viability benefits of multiple scenarios as an outcome of hypothetical reasoning and cognitive simulation. In fact, “we create temporary models of the world and test out actions (or alternative causes) in that simulated world” (Stanovich et al., 2011) by harnessing knowledge abstracted from previous experiences. Since type 1 processes are more or less confined to the here and now, type 2 processes need an independent structure to “decouple” (Stanovich et al., 2011) from it. Apparently working memory provides this simulation infrastructure.

In the Section, “Two Modes of Cognition,” we coupled the key differences of the co-creation and coping modes to the difference between ‘resilience’ and ‘stability.’ Reapplying this notion here suggests that type 1 processes use the resilience of the “generated” Umwelt as a quality measure so that increasingly resilient beneficial properties of the Umwelt are suggestive of desirable action outcomes. Similarly type 2 processes search for particular forms of stability and predictability; for example through discovering phenomena and their properties across many manifestations of the Umwelt.

Type 1 processes provide us with a rough sense of where we are, what is going on, and which acts will enhance or deteriorate the resilience of key components of the environment. Type 2 processes are the basis for explicit knowledge; in particular knowledge about the many interacting agents and processes that shape and define the Umwelt, its dynamics, and, via our acts, the world. By activating particular type 2 knowledge configurations as abstracted hypotheses of simulated Umwelt states, which feed back to type 1 processes as self-generated “input,” type 1 processes can associate resilience estimates. In fact it seems that the brain has an infrastructure for this that switches between sensory and self-generated “input” (Buckner et al., 2008).

Type 2 processing has been shown to correlate with general intelligence, while type 1 processing does not (Evans et al., 2010; Evans and Stanovich, 2013). We propose that ‘understanding’ is associated with the ability of type 1 processes to predict resilience effects: a new concept is ‘understood’ if its resilience effects can be predicted in an open world. One understands the world deeply if one can use the resilience and fragility in the world to reliable select actions that contribute to a favorable future.

This general description allows us to argue that a number of phenomena from different domains of psychology are actually manifestations of the interplay of type 1 and 2 processes as defined above. For example experiments addressing the time course of visual perception (Greene and Oliva, 2009) indicate that general, often action-selection related, landscape properties such as naturalness, depth, possibilities for concealment, and navigability can be estimated from a shorter image exposure than basic-level categorizations like forest, mountain, desert, and lake. We interpret this as type 1 processes setting up the stage for action selection and are therefore aimed at the activation of a situationally appropriate action repertoire through answering the questions “Where am I?” and “What is my default response?” Consecutively, type 2 processes augment this with knowledge abstracted across many different previous situations to interpret the situation better and to propose “better than default responses” back to type 1 processes for appraisal and comparison with expected sensory details.

A very similar account, at a longer timescale, can be formulated for emotion research, considering the common definition of emotion as ‘action readiness’ (Frijda, 1986). Emotion researchers make a difference between basic and complex emotions, where basic emotion arises directly as action readiness from the interplay between body and sensory stimulation (Izard, 2007). In contrast, complex emotions like emotional schemas “are defined in terms of the dynamic interaction of emotion and cognition” and “differ across individuals and cultures” (Izard, 2007). As such the actions they give rise to are not innate but learned from experience or imitation and thus they may represent vast amounts of tacit knowledge. Directly related to this distinction is the process of ‘emotion regulation’ (Gross and Thompson, 2007) in which deliberative processes change an initial emotion/action readiness into another more appropriate form. Both complex emotions and emotion regulation depend on the interplay between type 1 situational awareness, type 2 proposals for better than default outcomes, and type 1 evaluations of these proposals.

A third and last example involves mind wandering. It seems that people spend between 25 and 50% of their waking hours on thoughts unrelated to the here and now (Smallwood and Schooler, 2015). The ‘default mode network,’ directly associated with mind wandering, seems a fundamental function of the mammalian brain (Lu et al., 2012). “In the absence of an immediate need for goal-directed attention to the surrounding environment, our minds wander from recollection of past happenings to imagination of future events (Lu et al., 2012).” This can be interpreted as: when not in the coping mode, the mind wanders according to the dynamics of the co-creation mode (type 1), allowing the sequential reasoning about possibilities by type 2 processing, and performing resilience appraisal of these possibilities by type 1 responses.

The reported functions of mind wandering include prospection through simulating future activities, creativity via testing new solutions or perspectives, developing a meaningful life narrative, allowing for mental breaks, and to provide similar functions as dreaming (Smallwood and Schooler, 2015). A more abstract function, spanning decades and probably encompassing all reported functions, is the optimization of thought outcomes. Mind wandering, through its random nature, can be used to revisit, examine, and if need be improve, all knowledge and skills of a living agent and in doing so gradually upgrade one’s unexamined and more or less accidentally acquired ‘mental content 1.0,’ into a critically examined more empowering ‘mental content 2.0.’ This is what Perry (1998) describes as a key feature of the liberally educated mind and Van Rossum and Hamer (2010) mean by crossing the epistemological ‘watershed.’ Whatever it is called: it contributes, most effectively, to the agentic essence of optimizing future long-term viability through improved action selection.

### Example 3: Intelligence and Power versus Understanding and Wisdom

The concept of ‘understanding’ has emerged a number of times in this paper. Interestingly, well-developed understanding was always associated with the co-creation mode. Apparently well-developed understanding is not characteristic for the coping mode of cognition. However, due to the coping mode’s focus on the solution or mitigation of pressing problems, the concept of ‘intelligence’ is definitely a key feature of the coping mode of cognition. Well-developed intelligence, as measured by an IQ-test, *reflects the capacity to solve problems with known and fixed outcomes (which are therefore closed-world problems)*. This leads to the supposition that ‘intelligence’ manifests itself as the ability to solve or otherwise address pressing problems within a specific known domain.

In contrast, well-developed ‘understanding’ should, conforming with the logic of the co-creation mode, manifest itself as the *ability to create, maintain, and influence many aspects of one’s habitat with pervasive and long term flourishing as objective and measure-of-success.* Unlike intelligence, understanding is an open-world competence. Where intelligence is ideal for problem solving in known, fixed, and bounded contexts, understanding develops as one learns to grasp the general and invariant structures of unconstrained reality.

Coping is not only about solving problems, it is also about preventing an ill-understood world from spinning out of control, i.e., making it more stable and predictable. It is therefore about “the ability to produce intended outcomes”: the definition of ‘power’ as proposed by Bertrand Russell (Russell, 1938). In Learning Autonomy, we summarized Sternberg’s (1998) definition of wisdom as “the ability to produce broadly beneficial desired results while taking the full consequences of behavior on the habitat into account.” This suggests, in the context of this paper, to define power as “the ability to produce specific (often complexity reducing) intended results in a certain bounded environment without taking the full consequences of behavior into account.”

This then leads to two sets of concepts pertaining to the core cognitive processes related to how individuals create, maintain, or influence their habitat, i.e., how authoritative they are as individuals (Andringa et al., 2013). The set associated with the *coping mode* is deficiency or need driven and aims to exploit (to satisfy a pressing need) or to control (to reduce the complexity) the environment. In this mode ‘being authoritative’ means ‘exercising power’ and its key cognitive ability is ‘intelligence.’ The set associated with the *co-creation mode* is about the creation of a future in which it is easy to satisfy needs and as such it aims, via participation in, discovery of, using, promoting, relying on, and dynamically stabilizing the inherent dynamics of, the Umwelt in ways that maximize ‘resilience.’ In this mode ‘being authoritative’ equals being ‘wise,’ which requires a deep and pervasive understanding of the self and the Umwelt, manifested as the ability to produce broadly beneficial long-term results.

The lower block in **Figure 2** visualizes the relations between these concepts. To our knowledge, this is the first time that core concepts of (human) cognition are defined from first principles (namely ‘sustainability’ as defining property of life). That the terms ‘understanding’ and to a lesser extend ‘wisdom’ have received little scientific attention compared to ‘intelligence’ and ‘power’ is probably another sign of modern days’ narrow – coping mode associated – focus.

### Example 4: Haidt’s Moral Virtues

Haidt and Graham (2007) wrote a well-known article with the title “When Morality Opposes Justice: Conservatives Have Moral Intuitions that Liberals may not Recognize.” They argue that in the USA liberals typically recognize care (e.g., harm–prevention), and fairness as two key moral concerns. But, according to Haidt and Graham (2007):

Conservatives have many moral concerns that liberals simply do not recognize as moral concerns. When conservatives talk about virtues and policies based on the ingroup/loyalty, authority/respect, and purity/sanctity foundations, liberals hear talk about theta waves. For this reason, liberals often find it hard to understand why so many of their fellow citizens do not rally around the cause of social justice, and why many Western nations have elected conservative governments in recent years.

Why are liberals generally oblivious of the moral motivators of conservatives? We propose that liberals make moral judgments using only the logic of the co-creation mode of cognition, while conservatives do not fully trust on the outcomes of the co-creation mode and default to varying degrees of the coping mode logic. The result is that conservatives seem to use 2 + 3 = 5 moral virtues, while liberals rely on only two.

Haidt and Graham (2007) justify their five foundations of morality from an evolutionary point of view, but they do not go back further than mammalian care for young, and primate behaviors. We, of course, argue that the true foundations of the 2 + 3 = 5 pattern of moral virtues can be found in the unicellular level cooperation virtues that we summarized in **Table 1**. In this table we formulated two cooperation virtues (care and fairness) that aim to dynamically stabilize the environment through preventing individuals from slipping into the coping mode. In particular we noted: “empathy might therefore be understood in its original form as the ability to understand and be influenced by the state of the whole environment.” which translates as a concern for the state of and in particular the (potential) suffering of others and the environment in general.

However, for living agents that are in the coping mode we listed three more virtues for cooperation (ingroup loyalty, ingroup-role adherence, and self-imposed ingroup-rationality constraints) that allow ingroups to function as an effective and coherent whole. So we have a similar 2 + 3 = 5 pattern and indeed very similar sounding virtues. **Table 2** provides our interpretation of the moral virtues given the logic of the coping and the co-creation mode. Note that we interpret “harm/care” as generalized empathy, which we defined earlier as “the ability to understand and be influenced by the state of the whole environment.” Together with “fairness/reciprocity” this allows the implementations of “need inequality minimization” as key strategy of the co-creation mode.

**Table 2:** Haidt’s moral values (first column) compared to conservative and liberal morals.

Virtue	Interpretation	Conservative Coping mode	Liberal Co-creation mode
(1) Harm/care Basic concerns for the suffering of others, including virtues of caring and compassion.	Generic. Requires the ability to understand and be influenced by the state of the whole environment and the individuals in it.	Valued, but typically more for ingroups and on short and medium timescales, not a virtue extended to outgroups in times of anxiety.	Highly valued liberal key virtue, extended to unknown others, even in times of conflict.

(2) Fairness/reciprocity Concerns about unfair treatment, inequality, and more abstract notions of justice.	Generic. Requires understanding of adverse consequences of inequality.	Typically valued to prevent problems with unfair treatment of self or ingroup if not adequately justified. Not relevant for outgroups in times of anxiety.	Highly valued liberal key strategy, basis of mutual cooperation, extended to unknown others, even in times of conflict.

(3) Ingroup/loyalty Concerns related to obligations of group membership, such as loyalty, self-sacrifice and vigilance against betrayal.	Specific for (sub-)culture. Aimed at protection of one’s (sub-)culture	Valued because the ingroup is the only environment in which one is adequate. Protecting and safeguarding the group is a form of complexity curtailment.	Somewhat valued, however the (in)groups are not sacred and to be protected at all costs.

(4) Authority/respect Concerns related to social order and the obligations of hierarchical relationships, such as obedience, respect, and proper role fulfillment.	Specific for (sub-)culture. Aimed at complexity reduction through maximizing centrally controlled behavior.	Valued since authorities are the ones who are responsible for a personal feeling adequacy and social complexity management.	Somewhat valued, however the need for authority is indicative of an unnecessary dependency (a weakness to be overcome).

(5) Purity/sanctity Concerns about physical and spiritual contagion, including virtues of chastity, wholesomeness and control of desires.	Specific for (sub-)culture. Self-imposed complexity reduction through minimizing deviant and group-eroding behavior.	Valued virtue associated with norm adherence and especially resistance to temptations to violate norms.	Somewhat valued virtue, however, it should not prevent opportunities for exploration and growth.

The first column of **Table 2** provides Haidt’s and Graham’s moral virtues and their descriptions (Haidt et al., 2009). The second column gives our more generic interpretation of the moral virtues by connecting them to the cooperation virtues that we defined in **Table 1**. The third and fourth columns indicate the degree to which the moral virtues are valued given the logic of the conservative or coping mode and the logic of the liberal or co-creation mode.

In this fourth example we showed that unicellular level cooperation virtues seem to be, as we predicted on the basis of the Rule of Conservative Changes, fully preserved in the pattern of (human) moral behavior. And again the distinction between the coping mode and the co-creation mode is the defining factor.

### Example 5. The Role of Positive Emotions

The co-creation mode is associated with autopoietic success and, by extension, the co-creation mode of cognition in humans is associated with human thriving. Thriving is not a fixed or stable state of being. On the contrary, it is a dynamically developing process of succesfully fostering, cocreating, and maintaining relations between individuals and their environment to fully satisfy immediate and future needs alike. Yet for all its inherent complexity, reaching and maintaining thriving states should be the most natural thing to do: it is what life aims for, it is life’s measure of success. So what are the drivers and motivators of succesful living? One definite candidate is the set of positive moods and emotions. While negative moods and emotions are associated with states we want to avoid or end, positive moods and emotions are associated with states we actively seek or aim to perpetuate. This subsection investigates whether our understanding of positive emotions complies with the structure and role of the co-creation mode of cognition.

“Relative to negative emotions, positive emotions are few in number and rather diffuse (Fredrickson, 1998),” which makes sense because unlike the coping mode’s clear need satisfaction goals and focused activities, the co-creation mode is not immediately need driven, but associated with the discovery and maintenance of relations with other individuals and the habitat as a whole. We expected that the role of positive emotions could be framed in terms of the coping and co-creation mode, and indeed Fredrickson’s Broaden-and-Build Model of Positive Emotions (Fredrickson, 1998; Fredrickson and Branigan, 2005) does just that. In fact Fredrickson and Branigan (2005) frame negative and positive emotions surprisingly similar to our description of the coping and the co-creation mode. They write:

Whereas many negative emotions narrow individuals’ momentary thought-action repertoires by calling forth specific action tendencies (e.g., attack, flee), many positive emotions broaden individuals’ momentary thought–action repertoires, prompting them to pursue a wider range of thoughts and actions than is typical, e.g., play, explore, savor, and integrate (**Table 2**).

Whereas the narrowed thought–action repertoires of negative emotions were likely adaptive to our ancestors within specific threatening instances, the broadened thought–action repertoires of positive emotions were likely adaptive over the long-run. Broadened thought–action repertoires gain significance because they can build a variety of personal resources

This coheres our description of the core function of the co-creation mode that we described in **Figure 2** as “Discovering, using, promoting, and relying on the inherent dynamics of the environment (promoting resilience)” for which the development of understanding is characteristic. Positive emotions spur us to engage in our environment, to learn its properties, and stabilize it through participation. The description of the four positive emotions that Fredrickson and Branigan (2005) describe in detail complies with this. In **Table 3** we present representative quotes pertaining to ‘joy,’ ‘interest,’ ‘contentment,’ and ‘love’ and interpret the quotes in terms of the co-creation mode.

**Table 3:** Positive emotions and the co-creation mode.

Positive emotion	Description in relation to building and broadening of thought-action repertoire. All quotes from (Fredrickson, 1998)	Interpretation in terms of the co-creation mode.
Joy	*Joy, then, not only broadens an individual’s momentary thought–action repertoire through the urge to play, but also, over time and as a product of recurrent play, joy can have the incidental effect of building an individual’s physical, intellectual, and social skills.*	Play; exploring and learning to rely on the inherent dynamics of the environment.

Interest	*The momentary thought-action tendency sparked by interest, according to Izard (1977) is exploration, explicitly and actively aimed at increasing knowledge of and experience with the target of interest. Interest generates ”a feeling of wanting to investigate, become involved, or extend or expand the self by incorporating new information and having new experiences with the person or object that has stimulated the interest.”*	Discovering and exploring experiences and sources of knowledge in the zone of proximal development (cf Vygotskiĭ, 1978)

Contentment	*Contentment, one could argue then, is not simple passivity, but rather a mindful broadening of a person’s self-views and world views. Moreover, contentment appears to be the positive emotion that follows experiences that Csikszentmihalyi (1990) described as flow (described in connection with joy): “when the flow episode is over, one feels more ’together’ than before, not only internally but also with respect to other people and to the world in general.... The self becomes complex as a result of experiencing flow.”*	Process of consolidating newly discovered relations to extend the scope of understanding the living-environment

Love	*In the moment, exploring, savoring, and being playful with loved ones seems to have no obvious aim other than intrinsic enjoyment. Over time, however, the interactions inspired by love no doubt help to build and strengthen social bonds and attachment. These social bonds are not only satisfying in and of themselves, but are also likely to be the locus of subsequent social support. In this sense, love and the various positive emotions experienced in love relationships (i.e., interest, joy, and contentment) build and solidify an individual’s social resources.*	Developing and nurturing strong long-lasting bonds of trust and reliance to dynamically stabilize the (shared) environment.

Interestingly, in a more recent paper Cohn et al. (2009) study the term ‘ego-resilience’ which they describe as “a fairly stable personality trait that reflects an individual’s ability to adapt to changing environments.” They conclude, in complete agreement with our discourse:

Positive emotions are a powerful source of growth and change, predicting both individuals’ judgments about life and their skills for living well. […] it is not sufficient to appreciate or approve of one’s life in a general way; lived experiences such as joy and interest are what start the process of exploring, learning, connecting, and ultimately building new resources. Those resources can later improve one’s life, offering up new opportunities for enjoyment and resource building.

As is typical for Psychology, this deep insight, associated with resilience buildup, could have been generalized to the role of the co-creation mode of cognition as we have defined it. Yet it is not. Psychologists (and other specialist) have been trained not to venture outside of the bounds of their discipline. Which brings us back to the call topic.

## Prospects for a ‘Modern Synthesis’ in the Sciences of the Mind

*True, true, with no room for doubt, certain, worthy of all trust. See, the highest comes from the lowest, and the lowest from the highest; indeed a marvelous work of the tao. See how all things originated from it by a single process.*First three lines of a hypothetical original of the Emerald tablet of Hermes (Needham and Ping-Yu, 1980).

“As above so below” has been a valuable truth in esotericism and alchemy for many centuries. We have used the Rule of Conservative Changes to connect the “lowest and oldest” with the “highest and newest” and in doing so we have formulated life’s capacity to survive and thrive as the process that not only originates all of the biosphere, but that also defines human behavior. Starting from the unity of existence is, in our opinion, just as valuable today as was for the ancient minds that tried to understand the diversity of existence.

Starting from the unity of existence might be the only, and actually perfectly logical, method to avoid the fragmentation of knowledge so characteristic of modern day Psychology (Newell, 1973) and other fields of science. Yet the fragmentation of knowledge underlies the need for call topics like the current one: “Prospects for a ‘Modern Synthesis’ in the sciences of the Mind.” As we noted in example 1, our Western “left-hemispheric” – coping – world-view favors a narrow focus over the broader picture, specialists over generalists, fragmentation over unification, and knowledge and intelligence over experience and wisdom. For science this is also the case. To quote Einstein, “Problems cannot be solved with the same mind set that created them.”

Although we hope that this paper is an example of the strengths of the coping mode (i.e., the scientific method), it did essentially depend on the co-creation mode of cognition and more specifically on the positive emotions that guided us through the process. It was the *joy* of playing with the concepts and results of other thinkers that motivated us and kept us going in the absence of tangible results. It was our *interest* in phenomena just out of reach and in tantalizingly vague associations between disparate fields of science that gave us direction. And we felt *contentment* after hours of flow as a sign of achievement without us being able to specify what we actually had achieved. Finally, our *friendships* allowed us to be scathingly critical and supportive at the same time, to be patient with each other’s inability to formulate gut feelings in a clear manner, and turn this into a collaborative project.

Although not generally acknowledged, these positive emotions, motivations, and gut feelings are a normal part of science (Scheffer, 2014). They should become a *central* part of science if we really want to pool the insights and wisdom of (among others) scientists to allow us realistic “prospects for a conceptual synthesis or convergence of research focused on understanding mind and mindedness” (cf the call text).

What is probably not (yet) a normal part of science is our disrespect for arbitrary disciplinary boundaries. If the aim is a unity of science, it makes little sense to start with arbitrary (or opportunistic) disciplinary boundaries and then hope that one or a few new disciplines or research hypes will, uncharacteristically, not add to more fragmentation but lead to unification instead. Just as unlikely is some sort of “miracle” or super insight that allows us to mentally reconstruct a city by combining the rubble of more and more individual buildings.

Instead we argue for a complementary approach: a search for unity based on the essential and the invariant. We should start with the unity of existence and add detail only when we know how the details relate to the whole. Of course we do not know what is most essential and invariant. Yet, as the quote above suggests, we are also not fully unaware. What is really essential and invariant has influenced life in general and humanity in particular over its existence. The essential and the invariant define us and are as such coded deep in each of us. In fact our Western culture, for all that it brought us, might have obscured the essential and allowed us to live according to the logic of the coping mode while maintaining the illusion that we thrive and understand our existence (McGilchrist, 2010).

In this paper we used the Rule of Conservative Changes and the defining properties of life as invariant ‘truths” that allowed us to come up, among other connections, with the concept map in **Figure 2** that connects and specifies a number of core concepts of the behavioral sciences. While we expect that these two concepts are “pretty essential” and as such highly productive, we do not yet dare to claim that they go to the very core. This requires much more work, and probably reformulations of concepts and a sharpening of our reasoning. It needs a lot of reflecting and wrestling (playing actually) with results, insights, and hunches to make them all fit. Above all it requires the freedom and friendships to do so.

Finally, to answer the question that we started with: the origins of “the capacity to live life to the benefit of self and others” are not uniquely human. These originate in the defining properties of life and more explicitly in the inability of early life to evaluate its state separately from its environment. This “original perspective” allowed life to improve its own state by contributing to an easier-to-live-in environment and eventually to the creation of the biosphere. In humans this holistic understanding is preserved as empathy and wisdom. And although wisdom is still informing our ethical and political choices, it has to compete with pressing demands and the coping mode’s intelligent exploitation of environmental utility. Yet if intelligent power play wins too often it will destroy our environment as an adverse side effect. Only our “inability to separate us from our environment,” and the wisdom it leads to, can prevent this.

## Footnotes

1 Ghysen limited himself to the evolution of metazoa (all animals except protozoans and sponges). We apply the rule to all functions of life.
